# Tethered Cord Syndrome Secondary to the Unusual Constellation of a Split Cord Malformation, Lumbar Myelomeningocele, and Coexisting Neurenteric Cyst

**DOI:** 10.1155/2012/635029

**Published:** 2012-09-06

**Authors:** Humphrey Okechi, A. Leland Albright, Ancent Nzioka

**Affiliations:** ^1^Neurosurgeon, AIC Kijabe Hospital, P.O. Box 20, Kijabe 00220, Kenya; ^2^Neurosurgery Clinic, University of Wisconsin Hospitals and Clinics, Madison, WI 53792, USA; ^3^School of Health Sciences, Kenyatta University, Nairobi 00100, Kenya

## Abstract

We describe a seminal case report of a child with a tethered cord syndrome secondary to the unusual constellation of a split cord malformation, lumbar myelomeningocele, and coexisting neurenteric cyst. A 17-year-old adolescent girl with a several-month history of myelopathy and urinary incontinence was examined whose spinal MRI scan demonstrated a type II split cord malformation with a large bone spur and an intradural neurenteric cyst in addition to lumbar myelomeningocele. Untethering of the spinal cord was achieved via a lumbar laminectomy. Pathological examination confirmed the intradural cyst to be a neurenteric cyst. Postoperatively there was stabilization of the neurological symptoms. Prophylactic surgery with total resection of the neurenteric cyst when feasible and spinal cord un-tethering appears to be associated with excellent outcomes.

## 1. Introduction

Spinal neurenteric (NE) cysts are rare developmental malformations of the spinal cord. Although predominately encountered in the thoracic and cervical regions, they have also been reported, albeit uncommonly, in the lumbar region. In isolation NE cysts account for <1.5% of malformative spinal axis tumors [1]; however, NE cysts have also been reported in association with either split cord malformations or myelomeningoceles although never concurrently. Herein we present a seminal case detailing our experience treating a patient with a tethered cord syndrome secondary to the unusual constellation of a split cord malformation, lumbar myelomeningocele and coexisting NE cyst. A synopsis of the presentation and management of spinal NE cysts is also reviewed.

## 2. Case Presentation

### 2.1. History and Examination

A-17-year old adolescent girl came to our hospital with a 1-year history of episodic, progressively worsening back pain. She had also noticed increasing weakness and diminishing sensation in her lower extremities during this period and had recently developed urinary incontinence.

**Figure 1:**
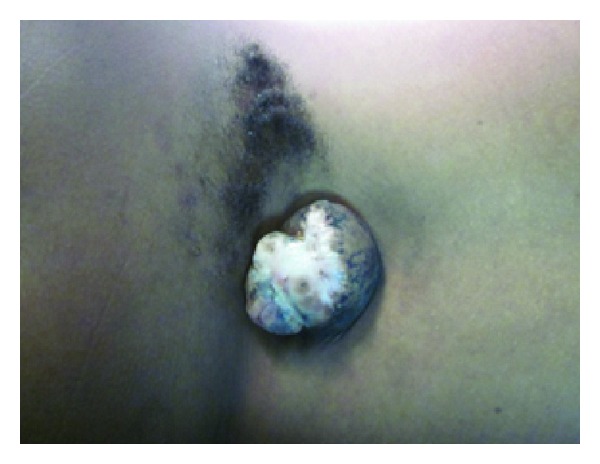
Lower lumber cicatrized myelomeningocele with hypertrichosis.

On physical examination, she had a cicatrized/epithelialized midline mass in the upper lumber region with hypertrichosis lateral to it, Figure 1. Her upper extremity motor function was normal but, in the lower extremities, she had no ankle dorsiflexion on either side, normal patellar reflexes, and absent Achilles reflexes.

**Figure 2:**
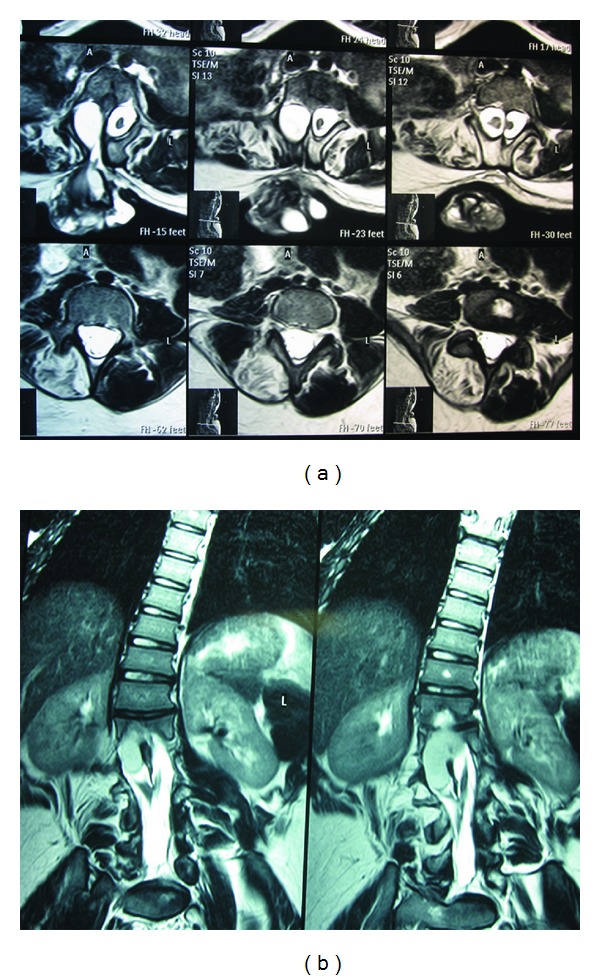
(a) Axial lumbar MRI T-2 images demonstrating a type 1 split cord malformation with the myelomeningocele attached to the right hemicord. (b) Coronal lumbar MRI T-2 images demonstrating a type 1 split cord malformation with a neurenteric cyst attached to the right hemicord.

MRI sagittal T2 images revealed a hypointense nonenhancing cystic mass with a low-lying conus, a type 1 spilt cord malformation with a bone spur as per Pang et al. classification [2], and a myelomeningocele attached to the right hemicord, Figures 2(a) and 2(b). A diagnosis of tethered cord secondary to mechanical tethering of the cord by the myelomeningocele and bone spur was made. Surgical intervention to untether the cord was recommended but was declined by the family. Six months later the patient returned with worsening lower extremity weakness—bilateral grade 2 hip flexion and grade 0 at the knee and ankle joints. She also had grade 4 right patella hyper-reflex.

### 2.2. Operative Findings

A laminectomy two levels proximal to the lesion was performed to expose the normal dura. Subsequently, the dura was opened followed by excision of the subcutaneous fibrotic mass. The right hemicord was then untethered from the dura. Aspiration of creamy pale grey material from the cyst was done before partially excising the cyst wall leaving the component that was adherent to the dorsal cord intact. The bone spur between the two dural sacs was resected and the dura closed primarily before a standard closure of the myocutaneous layers.

## 3. Histopathological Examination

**Figure 3:**
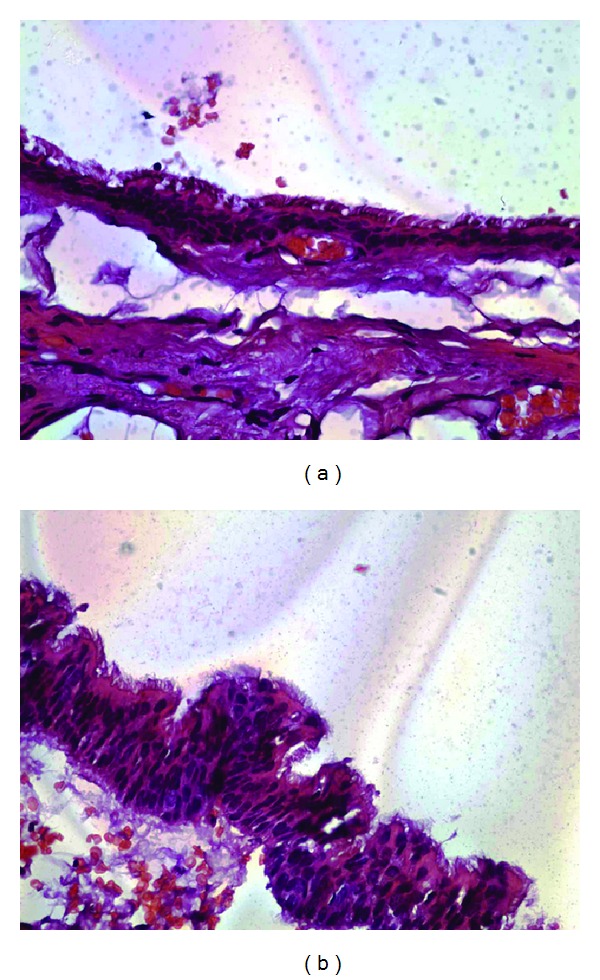
(a) Type A neurenteric cyst composed of respiratory epithelium with a basement membrane supporting pseudo stratified ciliated columnar epithelium at low magnification. (b) Type A neurenteric cyst composed of respiratory epithelium with a basement membrane supporting pseudo stratified ciliated columnar epithelium at high magnification.

Histopathological diagnosis of the lesion was described as type A neurenteric cyst (see Figures 3(a) and 3(b)).

**Postoperative Course**

Follow-up at 12 months postoperatively revealed stabilization of the neurological or urinary symptoms.

## 4. Discussion

The posited pathogenesis of neurenteric cysts by Dias and walker [3] establishes an association between neurenteric cysts and other anomalies of the spinal cord. Neurenteric cysts arise as a result of nondisjunction of the neuroectoderm from the endoderm during gastrulation, resulting in persistence of an entodermal-lined tract which may cause spina bifida or split cord malformations in addition to a myriad of other vertebral anomalies. Rauzzino et al. [4] in their series of 13 patients with neurenteric cysts found that all but one patient had some form of vertebral anomaly.

As solitary lesions neurenteric cysts are exceeding rare congenital developmental lesions, accounting for 0.5–1.3% of all spinal tumors [1]. Nevertheless, NE cysts have been reported in association with other spinal dysraphic states [5]. However, to the best of our knowledge this is the first case report of tethered cord syndrome secondary to concurrent existence of myelomeningocele, SCM, and neurenteric cyst.

The histopathology of neurenteric cysts has been described as a collection of mucin-producing simple columnar or cuboidal ciliated and nonciliated goblet cells surrounding a central cystic cavity. Neurenteric cysts are classified by the World Health Organization under the heading of “other malformative tumors and tumor-like lesions” and are described as cysts “lined by mucin secreting epithelium resembling that of the gastrointestinal tract” [6]. Wilkins subclassified NE cysts based on histological features of the cyst wall and its contents. The walls of type A cysts mimic gastrointestinal or respiratory epithelium with a basement membrane supporting single or pseudo stratified cuboidal or columnar cells, which may be ciliated. Type B cysts also contain glandular organization, usually producing mucin or serous fluid. Type C cysts are the most complex containing ependymal or glial tissue within the cyst [7].

However, the subclassification proposed by Wilkins has no correlation with the location or outcome of the NE cysts.

The majority of published cases of NE cysts in the literature suggest a predominant location in the thoracic and cervical spine, although lumbar cysts do occur. The higher incidence of NE cysts in the thoracic spine has been attributed to the embryonic origin of the neurenteric canal in the thoracic spine. Anatomically, NE cysts may be extradural, intradural extramedullary, or intradural intramedullary. The intradural, extramedullary location is encountered most commonly [2, 5].

Clinically, the most patients with NE cysts are diagnosed during the second decade of life and usually present with myelopathic symptoms. However, there have been reports of acute presentation with rapid onset of symptoms [4].

MRI is the diagnostic modality of choice in occult spinal dysraphism [5, 8, 9]. Neurenteric cysts on MRI scans appear as isodense lesions on T-1 images and hyperintense on T-2 images, without enhancement.

Surgical excision is the treatment of choice for most forms of occult spinal dysraphism. Although complete surgical excision of spinal neurenteric cyst is the ideal, it may not be possible if the cyst has an intramedullary component or is adherent to the neural tissues. In such situations, subtotal resection to minimize morbidity is an appropriate goal. Although partial resections carry a higher risk of recurrence, Garg et al. in a retrospective study of 23 patients followed up for a mean duration of 71 months found that partial resection was not associated with poorer outcomes [10].

We recommended untethering of the spinal cord when the patient first presented. Most series of patients with OSD have demonstrated neurological outcome to be related to the preoperative neurological status. Postoperatively 18% of patients will experience a deterioration or minimal improvement in their conditions [11, 12]. However, a rapid improvement in neurological function is to be expected in up to 70–80% of cases following cord un-tethering [10, 11]. In our case it is possible that earlier surgical intervention would have averted the neurological deterioration.

Recurrence rate of up to 37% has been reported following partial resection of NE cyst [13]. In a series of 16 patients with NE Santos De Oliveira et al. found recurrence in 3/4 patients after subtotal resection [12]. Although Cai et al. and Garg et al. reported lower rates of recurrence, the follow-up durations were relatively short, with means of 21 months and 38 months, respectively [10, 11]. In the series of 16 patients reported by Chavda et al., the duration between surgery and recurrence was 4–14 years [13]. Recurrence at such long intervals indicates a need for prolonged follow up and MR scanning after subtotal resections.

In conclusion, although entirely rare, concurrence of myelomeningocele, split cord malformation, and spinal neurenteric cyst is possible. Prophylactic surgery with total resection of the neurenteric cyst when feasible and spinal cord untethering should be undertaken prior to onset of neurological deficits.
